# Diagnostic accuracy of tests to detect hepatitis B surface antigen: a systematic review of the literature and meta-analysis

**DOI:** 10.1186/s12879-017-2772-3

**Published:** 2017-11-01

**Authors:** Ali Amini, Olivia Varsaneux, Helen Kelly, Weiming Tang, Wen Chen, Debrah I. Boeras, Jane Falconer, Joseph D. Tucker, Roger Chou, Azumi Ishizaki, Philippa Easterbrook, Rosanna W. Peeling

**Affiliations:** 1 London School of Hygiene and Tropical Medicine, Keppel St, London, WC1E 7HT UK; 2 Guangdong Provincial Center for Skin Diseases and STI Control, Guangzhou, China; 3 School of Medicine, University of North Carolina at Chapel Hill, Chapel Hill, NC USA; 4 School of Public Health, Sun Yat-sen University, Guangzhou, China; 5 Oregon Health & Science University, Portland, OR USA; 6 HIV/ AIDS Department, World Health Organisation, Geneva, Switzerland

**Keywords:** Diagnostic accuracy, Diagnostic tests, Hepatitis B virus, Rapid diagnostic tests, Enzyme immunoassays, CMIA, MEIA

## Abstract

**Background:**

Chronic Hepatitis B Virus (HBV) infection is characterised by the persistence of hepatitis B surface antigen (HBsAg). Expanding HBV diagnosis and treatment programmes into low resource settings will require high quality but inexpensive rapid diagnostic tests (RDTs) in addition to laboratory-based enzyme immunoassays (EIAs) to detect HBsAg. The purpose of this review is to assess the clinical accuracy of available diagnostic tests to detect HBsAg to inform recommendations on testing strategies in 2017 WHO hepatitis testing guidelines.

**Methods:**

The systematic review was conducted according to the Preferred Reporting Items for Systematic Reviews and Meta-analyses (PRISMA) guidelines using 9 databases. Two reviewers independently extracted data according to a pre-specified plan and evaluated study quality. Meta-analysis was performed. HBsAg diagnostic accuracy of rapid diagnostic tests (RDTs) was compared to enzyme immunoassay (EIA) and nucleic-acid test (NAT) reference standards. Subanalyses were performed to determine accuracy among brands, HIV-status and specimen type.

**Results:**

Of the 40 studies that met the inclusion criteria, 33 compared RDTs and/or EIAs against EIAs and 7 against NATs as reference standards. Thirty studies assessed diagnostic accuracy of 33 brands of RDTs in 23,716 individuals from 23 countries using EIA as the reference standard. The pooled sensitivity and specificity were 90.0% (95% CI: 89.1, 90.8) and 99.5% (95% CI: 99.4, 99.5) respectively, but accuracy varied widely among brands. Accuracy did not differ significantly whether serum, plasma, venous or capillary whole blood was used. Pooled sensitivity of RDTs in 5 studies of HIV-positive persons was lower at 72.3% (95% CI: 67.9, 76.4) compared to that in HIV-negative persons, but specificity remained high. Five studies evaluated 8 EIAs against a chemiluminescence immunoassay reference standard with a pooled sensitivity and specificity of 88.9% (95% CI: 87.0, 90.6) and 98.4% (95% CI: 97.8, 98.8), respectively. Accuracy of both RDTs and EIAs using a NAT reference were generally lower, especially amongst HIV-positive cohorts.

**Conclusions:**

HBsAg RDTs have good sensitivity and excellent specificity compared to laboratory immunoassays as a reference standard. Sensitivity of HBsAg RDTs may be lower in HIV infected individuals.

**Electronic supplementary material:**

The online version of this article (10.1186/s12879-017-2772-3) contains supplementary material, which is available to authorized users.

## Background

An estimated 257 million individuals worldwide are chronically infected with hepatitis B virus (HBV), of whom 2.7 million are co-infected with HIV [1]. Globally, between 20 and 30% of patients with chronic HBV infection will develop cirrhosis or hepatocellular carcinoma [2], accounting for the majority of the attributable 686, 000 deaths [3] and 21 million disability-adjusted life-years annually [4]. Most individuals with chronic HBV infection however are not aware of their serostatus. Delayed diagnosis means that many may progress to long term complications and present only with advanced disease [5]. Expanded access to testing for HBV is critically important in order to increase numbers of infected individuals aware of their status for linkage to care, as well as identifying candidates for HBV vaccination and facilitating prevention and control efforts.

In March 2015 the World Health Organization (WHO) published the first global guidelines for the prevention, care, and treatment of individuals with chronic HBV infection [5]. These guidelines focused on assessment for treatment eligibility, initiation of first and second-line therapies, and monitoring. These initial guidelines did not include testing recommendations, and in particular which tests to use. Given the large burden of HBV in low and middle income settings where there are limited or no existing HBV testing guidelines, the development of HBV testing guidelines is a priority.

Advances in HBV detection technology have created new opportunities for testing, referral, and treatment. Chronic HBV infection is defined as persistence of hepatitis B surface antigen (HBSAg) for at least six months, and the testing strategy involves an initial serological test to detect HBsAg followed by nucleic-acid amplification test (NAT) for detection of HBV DNA viral load to help guide treatment decisions [5]. HBsAg can be detected using rapid diagnostic tests (RDTs) in lateral flow, flow through or simple agglutination assays formats. Laboratory-based immunoassays to detect HBsAg include traditional radioimmunoassays (RIA) and enzyme immunoassays (EIA), as well as newer technologies such as electrochemiluminescence immunoassays (ECLIA), microparticle enzyme immunoassays (MEIA) and chemiluminescent microparticle immunoassays (CMIA), which use signal amplification to give quantitative measurements.

Previous systematic reviews on HBV infection have focused on effectiveness of immune responses to HBV vaccination [6], surveillance of cirrhosis [7], and evaluation of treatment effectiveness [8]. Prior reviews on hepatitis B testing [9–11] only focused on the performance of tests that can be used at the point of care. They also included evaluations with unclear reference standards and studies that used serum panels to evaluate test performance, which are inappropriate for assessing clinical or operational diagnostic accuracy in the field. This review aimed to assess the diagnostic accuracy of assays used to detect HBsAg in order to inform WHO and other guidelines on hepatitis testing [12]. This was the first study exclusively comparing the clinical performance of both RDTs and laboratory-based immunoassays, in addition to addressing the question of accuracy in the context of HIV status. The accuracy of HBsAg assays against a NAT reference standard was also undertaken, given the importance of reducing transmission during the seroconversion period and in the diagnosis of occult hepatitis B where HBsAg may not be detectable, which is more common with HIV co-infection. The purpose of this review was to provide quantitative evidence of the accuracy of available diagnostics to detect HBsAg in order to inform global guidelines.

## Methods

### Search strategy and identification of studies

We conducted a systematic review and meta-analysis on the diagnostic accuracy of HBsAg tests. The review was registered in PROSPERO (CRD42015020313) and reported in accordance with the Preferred Reporting Items for Systematic Reviews and Meta-analyses (PRISMA) check list. We utilised standardised methods for systematic reviews on diagnostics, including an *a priori* protocol (Additional file 1).

Literature search strategies were developed by a medical librarian with expertise in systematic review searching, using a search algorithm consisting of terms for: hepatitis B, diagnostic tests, and diagnostic accuracy. We searched MEDLINE, EMBASE, the Cochrane Central Register of Controlled Trials, Science Citation Index Expanded, SCOPUS, Literatura Latino-Americana e do Caribe em Ciências da Saúde (LILACS), WHO Global Index Medicus, WHO’s International Clinical Trials Registry and the Web of Science. We also contacted researchers, experts and authors of major trials, with no relevant manuscripts in preparation identified. Additional pertinent citations were identified through bibliographies of retrieved studies.

Abstracts were screened by reviewers AA and HK according to standard inclusion and exclusion criteria. All studies identified for full manuscript review were assessed independently by two reviewers (AA and OV) against inclusion criteria. Papers were accepted or rejected, with reasons for exclusion specified. Discrepancies were resolved by discussion between review authors and, when required, a third independent reviewer (RP).

### Selection criteria

Inclusion criteria were: case-control, cross-sectional, cohort studies or randomized trials published between 1996 and May 2015; primary purpose of evaluating HBsAg test accuracy; commercially available laboratory immunoassays or NAT as reference standard; any clinical specimen type. We excluded: articles in languages other than English; conference abstracts, comments or review papers; studies only reporting sensitivity or specificity without reference standards; studies using commercially prepared reference panels.

We included studies reporting original data from patient specimens in all age groups, settings, countries and specimen types. We performed a sub-analysis comparing test accuracy before 2005 with more recent studies published between 2005 and 2015 as the accuracy of reference standard immunoassays has improved over time. This time period was chosen as it was 10 years prior to the literature search, matched with a similar meta-analysis on hepatitis C tests (Ref Paper 11), and was around the time of the last WHO review of HbsAg assay operational characteristics [13]. Studies comparing the accuracy of laboratory based immunoassays were only included if they used CMIAs as the reference standard; most excluded studies using other platforms included reference panels, while five specifically used non-CMIA reference assays. Given the association between false negatives and a low OD/CO, it was reasonable to presume sensitivity is reduced with low HBsAg levels. CMIA has excellent analytical sensitivity (0.05 IU/ml) [14–16], and can be used to quantitate HBsAg levels in clinical specimens [17]. These platforms are the most widely used in clinical practice [18] given automation and high throughput, with data on kinetics and sensitivity in HIV-HBV co-infection.

### Data extraction and quality assessment

Two authors (AA and OV) independently extracted data and reached agreement on the following variables: study author and year; study location and design; specimens tested; eligibility criteria; index test and reference standard, including manufacturer; raw cell numbers (true positives, false negatives, false positives, true negatives); HIV co-infection; sources of funding and reported conflict of interest.

Study quality was evaluated using the QUADAS-2 tool [19], which evaluates risk of bias (patient selection, index test, reference standard, and patient flow through) and applicability concerns (patient selection, index test, reference standard).

### Data analysis and synthesis

We conducted meta-analysis pooling data using the DerSimonian-Laird bivariate random effects model (REM) to calculate pooled sensitivity and specificity with 95% confidence intervals (CI), which were used to estimate positive and negative likelihood ratios (PLR, NLR). Heterogeneity was assessed by visual inspection of forest plots and estimates of τ2 for diagnostic odds ratios (DOR) to measure between study variability. We performed sub-group analysis based on study year (2005–2015); tests brands (for brands that were evaluated in at least three studies); sample type and HIV status. All statistical analysis and figures were generated using Meta-Disc© version 1.4.7. (XI Cochrane Colloquium Barcelona, Spain).

## Results

### Study selection and characteristics

**Fig. 1:**
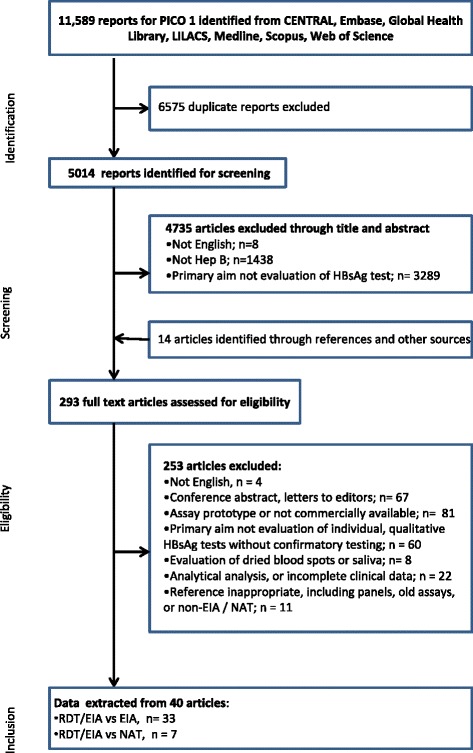
PRISMA flow Diagram of included studies

A total of 11,589 citations were identified, and 293 full-text articles examined which identified 40 studies meeting pre-defined criteria (Fig. 1). Of the included studies, 33 compared RDTs [14, 18, 20–47] and/or EIAs [14, 47–50] against an immunoassay reference standard, of which five focused on accuracy in HIV-positive individuals [26, 44–47]. Seven studies compared RDTs [51–53] and/or EIAs [53–57] against a NAT reference standard, of which 3 had data from HIV-positive patients [53, 56, 57]. Studies were all either cross-sectional or case-control, predominantly in the laboratory setting, and performed in a broad range of populations, including healthy volunteers, blood donors, pregnant women, incarcerated adults, HIV and hepatitis patient cohorts with confirmed HBV infection. The prevalence of HBV ranged from 1.9 to 84% in populations tested. A mixture of serum, plasma and whole blood was used for RDTs, while studies of EIAs were performed on serum or plasma samples. Study characteristics are presented in Tables 1, 2 and 3.

**Table 1:** Study characteristics of laboratory-based immunoassays against laboratory reference standard [EIA vs EIA]s

Study [author, year]	Location [country, city]	Sample size	Study design	Setting	Sample type	Assay under evaluation [type, brand]	Reference standard [type, brand]
Liu, 2014 [14]	China	250	CC	Hospital patients; outpatients (Preselected based on CMIA quantitative results)	Serum	ECLIA, Cobas ELISA, Wantai	CMIA, Architect HBsAg
Peng, 2011 [49]	China	498	CC	Hospital patients (Preselected based on S/CO from KHB screen)	Serum	ELISA, KHB	CMIA, Architect HBsAg
Geretti, 2010 [47]	Ghana, Kumasi	838	CS – CSQ	HIV clinic (1/3 on lamivudine)	Serum	CMIA, Architect HBsAg CMIA, Liaison Ultra EIA, Murex v3	CMIA, Architect/ Liaison EIA, Murex v3^a^
Ol, 2009 [48]	Cambodia	120	CS – CSQ	Blood donors (rural community)	Serum	ELISA, Monolisa	CMIA, Architect HBsAg
Viet, 2012 [50]	Vietnam	119	CS – CSQ	Blood donors (rural community)	Serum	EIA, Monolisa Ultra	CMIA, Architect HBsAg

^a^Reactive all three assays OR reactive in one assay with neutralisation

*CC* case control, *CMIA* chemiluminescent microparticle enzyme immunoassay, *CS* cross-sectional, *CSQ* consecutive patients, *ECLIA* electrochemiluminescent immunoassay, *EIA* enzyme immunoassay, *ELISA* enzyme linked immunosorbent assay, *KHB* KHB Ltd., Shanghai, *S/CO* signal cut-off ratio

**Table 2:** Study characteristics of rapid-diagnostic tests (RDTs) against laboratory reference standards [RDT vs EIA]

Study [author, year]	Total participants, n (substudy size, n)		Location [country, city]	Study design	Setting	Sample	RDT under evaluation [type, brand]	Reference test [type, brand]
Mvere, 1996 [36]	206		Zimbabwe	CS	Blood Bank	S	Dipstick (PATH) SimpliRed	EIA, Auszyme
Sato, 1996 [42]	462		Japan	CC	Hospital	S	Dainascreen Serodia	EIA, Auszyme
Abraham, 1998 [20]	450	(50)	India, Vellore	CC– Panel	Hospital patients (Multiply transfused; chronic liver disease; preop and antenatal patients)	S	QuickChaser Virucheck	EIA, Auszyme or Hepanostika
		(400)		CS– Screen				
Oh, 1999 [38]	250		Korea	CC - Panel	Blood donor panel	S	Genedia Serodia	EIA, Cobas Core
Kaur, 2000 [30]	2754		India	CS - CSQ	Hospital Surgery patients; blood donors; patients ruling out HBV	S	Hepacard	EIA, Ortho 3rd generation
Lien, 2000 [33]	328	(328)	Vietnam	CC	High risk volunteers High-risk volunteers; pregnant women; patients with other infectious diseases (including 10 with HIV); preselected HBsAg pos (101), HBsAg neg (99)	SP	Dainascreen Determine Serodia	EIA, Monolisa MEIA for discordant
		(128)		CS		S P WB	Determine	
Raj, 2001 [40]	999		India, Vellore	CS	Hospital laboratory samples (emergency preop screening; antenatal women in labour; haemodialysis; urgent donor screening)	S	Hepacard	EIA, Auszyme MEIA, AxSYM v2
Clement, 2002 [27]	942		Belgium	CC	Hospital - Patients with biopsy proven HBV; healthy volunteers from a vaccine evaluation trial; blood donors	WB, S	BinaxNOW	MEIA, AxSYM v2
Lau, 2003 [32]	2463	(1011)	USA	CS - CSQ	Hepatology clinics	S fresh	BinaxNOW	EIA, ETI-MAK2
		(827)		CS	Incarcerated offenders	S frozen		
		(625)		CS - CSQ	Chinese Community Health Fair (random patients); known HBV-positive patients (liver clinic)	WB		
Akanmu, 2006 [21]	137	(101)	Nigeria, Lagos	CS - CSQ	Blood donors (male)	WB	BinaxNOW	ELISA, Monolisa
		(36)			Chronic liver disease			
Nyirendra, 2008 [37]	194		Malawi	CS - CSQ	Hospital Hospital patients including 152 HIV+	P	Determine	EIA, Bioelisa Neutralisation (positives)
Lin, 2008 [34]	1250	(671)	China	CC	Blood donors (500); Clinical specimens HBsAg + (171)	S	Determine DRW	EIA, Hepanostika Ultra
		(579)	Guinea	CC	Blood donors (491); Stored positives (88)	SP		
Randrianirina, 2008 [41]	200		Madagascar	CC	Not specified	S	Cypress Determine Hexagon Virucheck	EIA, AxSYM
Ola, 2009 [39]	80	(25)	Nigeria	CS - CSQ	Medical clinic	WB	AMRAD GWHB	ELISA, Wellcozyme Kit
		(55)			Blood donors	S	Biotec Latex	
Khan, 2010 [31]	57		Pakistan	CC	NS	S	Accurate Onecheck	ELISA, 2nd generation
Davies, 2010 [26]	75		Malawi	CS - CSQ	HIV-positive adults (ART naïve)	S	Determine	EIA, Biokit Neutralisation (positives)
Bjoerkvoll, 2010 [22]	2400	(1200)	Cambodia	CS - CSQ	General screen - Blood donors (rural)	S	ACON	EIA, Monolisa Ultra^a^
		(1200)	Vietnam					
Geretti, 2010 [47]	838		Ghana, Kumasi	CS - CSQ	HIV-clinic (1/3 lamivudine)	S	Determine VIKIA	CMIA, Architect/ Liason EIA, Murex v3
Hoffman, 2012 [45]	973		South Africa	CS - CSQ	HIV-positive adults (ART naïve) - Antenatal or primary care	WB (cap)	Determine	ELISA, AxSYM
Bottero, 2013 [23]	3956	(2472)	France, Paris	CS - CSQ	General Screening (healthcare centres) [General population prevention, screening, vaccination]	WB(ven)	Determine	ELISA, Monolisa Ultra Neutralisation (positives)
		(3922)					QUICK PROFILE	
		(3928)					VIKIA	
Chameera, 2013 [24]	50		Sri Lanka	CS	Hospital (surgical, orther)	S	Cortez Onsite	EIA, Surase B-96 (TMB)
Franzeck, 2013 [44]	272		Tanzania, Ifakara	CS - CSQ	HIV-clinic (ART naïve)	WB(ven) P	Determine	EIA, Murex v3 Neutralisation (positives)
Chevaliez, 2014 [25]	1768	(558)	Various	CC	Chronic Hep B (known mutants, blood donors); HBsAg negative (mix, including HIV, 34; HCV, 48)	SP	DRW v2.0	CMIA, Architect
		(408)		CS	Acute hepatitis			
		(802)		CS	Pregnant - women at delivery			
Erhabor, 2014 [28]	130		Nigeria, Sokoto	CC	Blood donors	SP	ACON	ELISA, HBsAg Ultra
Gish, 2014 [29]	297		Australia, Melbourne	CS - CSQ	At risk Health fairs, outreach; Vietnamese (72%)	S	Nanosign	EIA, Quest Diagnostics
Honge, 2014 [46]	438		Guinea-Bissau	CS - CSQ	HIV clinic - mixed ART/ naïve	S	VEDA LAB	CMIA, Architect
Liu, 2014 [14]	250		China	CC	Hospital patients; outpatients (Preselected based on CMIA quantitative results)	S	Intec One Step	CMIA, Architect
Mutocheluh, 2014 [35]	150		Ghana	CS - CSQ	Blood donors	P	Abon Acull-Tell Core TM Rapid care Wondfo	ELISA, Human Gesellschaft
Upretti, 2014 [43]	347		Nepal	CS - CSQ	Children - pre and post vaccination; mothers (8)	S	SD Bioline	EIA, Surase B-96 (TMB)
Njai, 2015 [18]	1000	(178)	Gambia	CS	Hepatitis patients CHB carriers (study 3), incl 3 co-infected HIV (treatment naïve)	S	Determine	CMIA (quantitative), Architect
		(203)					Espline	
		(773)		CS - CSQ	General Community Screen	WB	Determine	EIA (DBS), AxSym + Neutralisation
		(476)					VIKIA	

^a^Validated random sample with CMIA, Abbott

*ART* antiretroviral therapy, *CC* case-control study, *CHB* chronic hepatitis B, *CMIA* chemiluminescent microparticle enzyme immunoassay, *CS* cross-sectional study, *CSQ* consecutive patients, *DBS* dried blood spot, *ECLIA* electrochemiluminescent immunoassay, *EIA* enzyme immunoassay, *ELISA* enzyme linked immunosorbent assay, *HBV* hepatitis B virus, *HCV* hepatitis C virus, *HIV* human immunodeficiency virus, *MEIA* microparticle enzyme immunoassay, *S* serum, *P* plasma, *WB (cap)* capillary whole blood, *WB (ven)* venous whole blood

**Table 3:** Study Characteristics of rapid-diagnostic tests (RDTs) or laboratory-based immunoassays (EIA) against nucleic-acid test reference standards [RDT/EIA vs NAT]

Study [author, year]	Location [country, city]	Sample size, n	Study design	Setting	Sample	Test under evaluation [type, brand]	Reference test [type, brand]
Ansari, 2007 [51]	Iran, Urumieh	240	CC	Hospital patients	S	RDT, ACON RDT, Atlas RDT, Blue Cross RDT, Cortez RDT, DIMA RDT, Intec	qPCR
Nna, 2014 [52]	Nigeria	113	CS	Blood donors (repeat) – all were HIV negative	P	RDT, ACON	Nested PCR; qPCR for positive
Seremba, 2010 [53]	Uganda	157	CS - CSQ	Hospital patients (emergency medical ward), including HIV+ (83) and HIV- (74).	S	RDT, Cortez EIA, ADVIA	PCR
Khadem-Ansari, 2014 [54]	Iran, Urumieh	350	CS – CSQ?	Hospital patients - referred as? HBV	S	ChLIA, Liaison	Rt-PCR
Lukhwareni, 2009 [56]	South Africa	192	CC	HIV cohort - pre ART	S	ChLIA, Elecsys	qPCR
Mphahlele, 2006 [57]	South Africa	295	CC	Hospital patients, HIV+ (167) and HIV- (128)	S	EIA, AxSYM	Nested PCR
Olinger, 2007 [55]	Nigeria, Ibadan	200	CS	Hospital patients - Liver disease, HIV	S	MEIA, AxSYM v2 ChLIA, Elecsys ELFA, VIDAS Ultra	rtPCR and nested PCR

*CC* case control, *ChLIA* chemiluminescent immunoassay, *CMIA* chemiluminescent microparticle enzyme immunoassay, *CS* cross-sectional, *CSQ* consecutive patients, *ECLIA* electrochemiluminescent immunoassay, *EIA* enzyme immunoassay, *ELFA* enzyme linked fluorescent assay, *ELISA* enzyme linked immunosorbent assay, *MEIA* microparticle enzyme immunoassay, *qPCR* quantitative PCR, *RDT* rapid diagnostic test, *rtPCR* realtime PCR

### Assessment of the quality of the studies

**Fig. 2:**
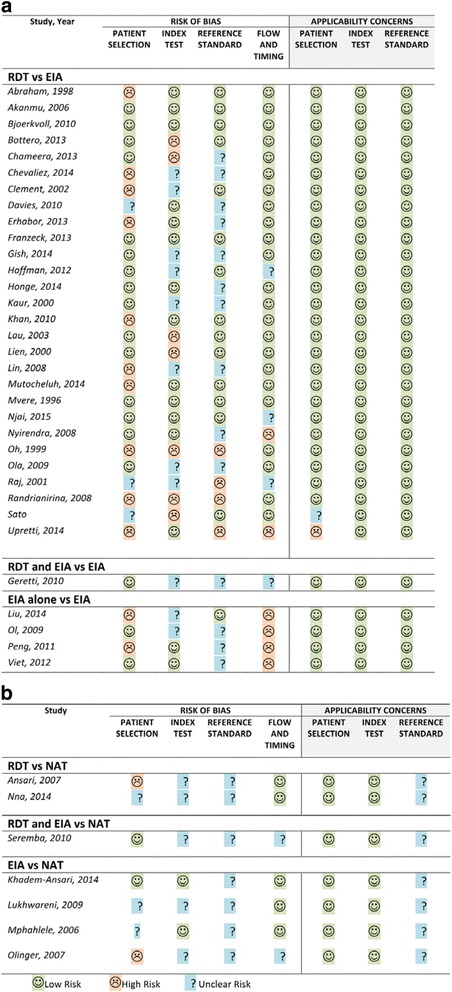
Risk of bias and applicability for studies using (**a**) laboratory, or (**b**) nucleic-acid reference standard

**Fig. 3:**
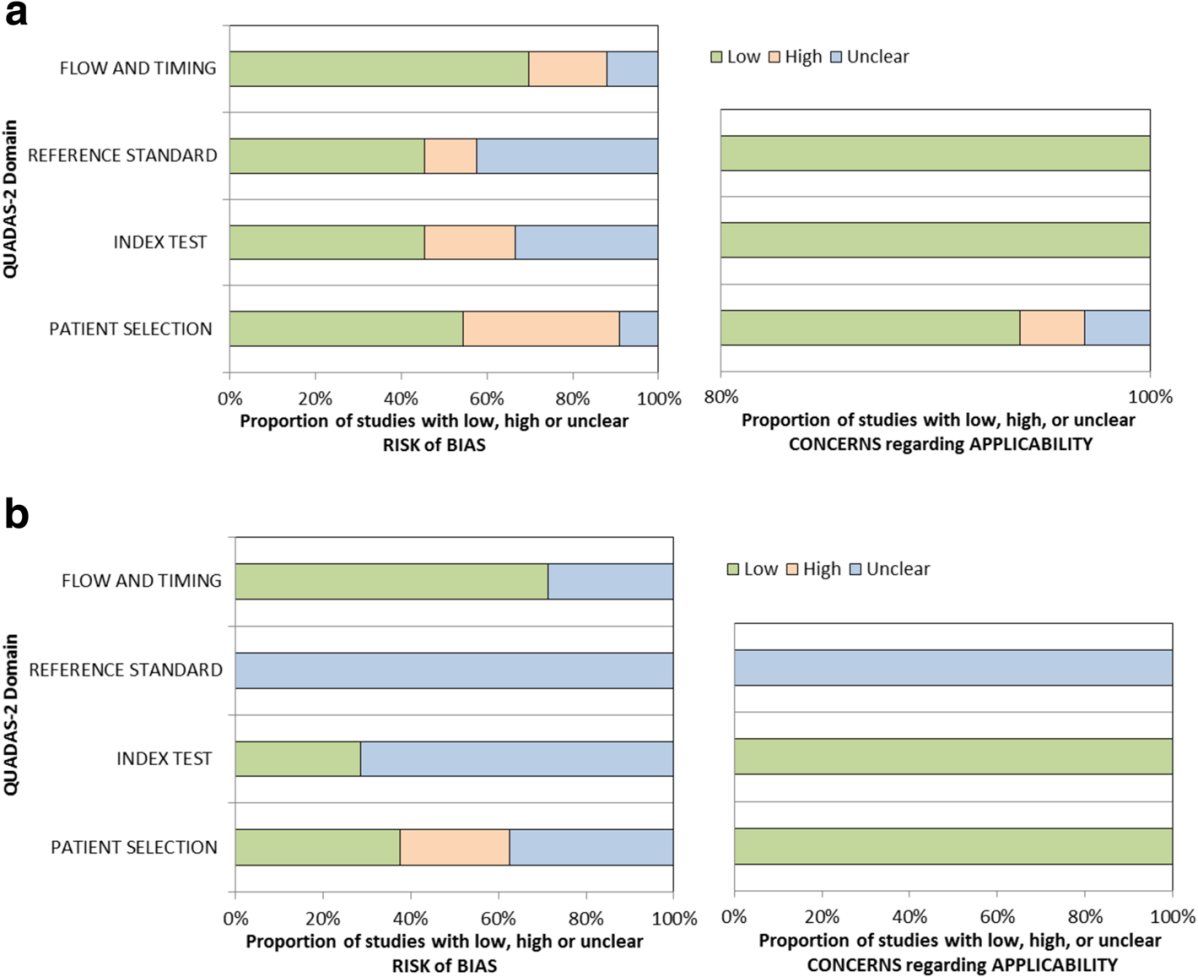
Risk of bias and applicability summary for using (**a**) laboratory, or (**b**) nucleic-acid reference standard

The QUADAS-2 assessment for risk of bias of each study, including sub-studies deriving separate data points is presented in (Fig. 2a, b), with a summary in (Fig. 3). Bias in patient selection was generally attributable to a case-control study design (38%), or from enrolment of highly selected populations such as blood donors or those with known hepatitis B virus infection. Risk of bias from the index test was most commonly due to insufficient reporting of blinding or evaluation of RDTs which are no longer commercially available. Although the majority of studies did not specify the exact time interval between performance of the index and reference assays, it was assumed to be at low risk of bias as the assays were performed on the same sample. Applicability was judged to be higher risk for bias predominantly due to inclusion of older studies, those that evaluated tests which are no longer commercially available or studies using a NAT reference.

### Diagnostic accuracy of rapid tests for HBsAg detection

**Fig. 4:**
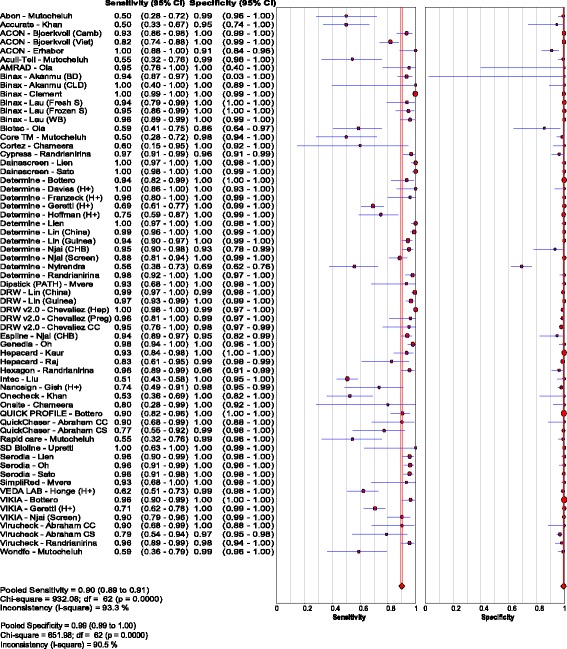
Forest plot with accuracy of RDT compared to EIA

Thirty studies [14, 18, 20–47] assessed the accuracy of 33 different brands of RDTs in 23,716 individuals, which resulted in 63 data points for sensitivity and specificity. The reference standards used were CMIA in 5 studies, MEIA in 3 studies, and EIA/ELISA in 25 studies, with 3 studies using more than one type of reference assay. Test evaluations were conducted in 23 countries: six studies were conducted in high-income country studies [23, 27, 29, 32, 38, 42], two in upper-middle income country studies [14, 34], nine in lower-middle income [21, 24, 28, 30, 31, 33, 35, 39, 47], and six in low income [18, 20, 22, 40, 43, 46] countries, with income levels classified according to the World Bank ranking criteria. The overall pooled sensitivity and specificity were 90.0% (95% CI: 89.1, 90.8) and 99.5% (95% CI: 99.4, 99.5), respectively. The positive and negative likelihood ratios were 117.5 (95% CI: 67.7, 204.1) and 0.10 (95% CI 0.07, 0.14), respectively. Visual and statistical heterogeneity (τ2 = 6.84) was present for pooled analyses of sensitivity and specificity; however, the range in sensitivity values (0.50 to 1.00) was much broader than the range in specificity values (0.86 to 1.00 in all studies except for 1) [Fig. 4 **;** Tables 2, 4 and 5].

**Table 4:** Summary pooled diagnostic accuracy of HBsAg assays using EIA reference standards

Index test	HIV status	Studies, n	Sample size (range), n	Data points, n	Pooled clinical accuracy (95% CI)		Likelihood ratios (95% CI)	
					Sensitivity	Specificity	Positive	Negative
RDT	NA	30	23,716 (25–3928)	63	90.0 (89.1–90.8)	99.5 (99.4–99.5)	117.5 (67.7–204.1)	0.10 (0.07–0.14)
	HIV-positive	5	2596 (75–973)	6	72.3 (67.9–76.4)	99.8 (99.5–99.9)	192.6 (77.4–497.2)	0.29 (0.22–0.38)
EIA	NA	5	1825 (119–838)	8	88.9 (87.0–90.6)	98.4 (97.8–98.8)	46.8 (12.9–170.0)	0.04 (0.01–0.13)
	HIV-positive	1	838	3	97.9 (96.0–99.0)	99.4 (99.0–99.7)	167.3 (95.1–294.1)	0.02 (0.01–0.04)

**Table 5:** Summary pooled diagnostic accuracy of rapid HBsAg assays stratified by study, patient, index and reference tests

	Subgroup	Studies, n	Sample size, n	Points, n	Pooled clinical accuracy (95% CI)		Likelihood ratios (95% CI)	
					Sensitivity	Specificity	Positive	Negative
Study	Pre 2005	9	8854 (206–2754)	19	96.9 (96.0–97.7)	99.7 (99.6–99.8)	265.5 (106.1–664.5)	0.06 (0.03–0.10)
	Post 2005	21	14,862 (25–3956)	44	86.4 (85.2–87.5)	99.4 (99.2–99.5)	84.6 (43.6–164.6)	0.13 (0.09–0.18)
	HIV-positive	5	2596 (75–973)	6	72.3 (67.9–76.4)	99.8 (99.5–99.9)	192.6 (77.4–497.2)	0.29 (0.22–0.38)
Specimen type	Whole blood	8	6889 (25–3956)	11	91.7 (89.1–93.9)	99.9 (99.8–99.9)	346.6 (157.6–762.4)	0.09 (0.06–0.14)
Index test	Determine	10	7730 (75–2472)	12	90.8 (88.9–92.4)	99.1 (98.9–99.4)	239 (17.1–33,300)	0.077 (0.035–0.168)
	VIKIA	3	5242 (476–3928)	3	82.5 (77.5–86.7)	99.9 (99.8–100)	1070 (376–3060)	0.108 (0.026–0.458)
	BinaxNOW	3	3542 (137–2463)	6	97.6 (96.2–98.6)	100 (99.7–100)	221 (36.1–1350)	0.045 (0.016–0.128)
	Serodia	3	1040 (250–462)	3	95.8 (93.4–97.5)	99.8 (99.1–100)	285 (71.4–1140)	0.045 (0.029–0.069)
Reference test	CMIA	5	3521 (227–1768)	9	80.4 (77.9–82.6)	99.0 (99.6–99.3)	58.5 (31.3–109)	0.141 (0.074–0.268)

**Fig. 5:**
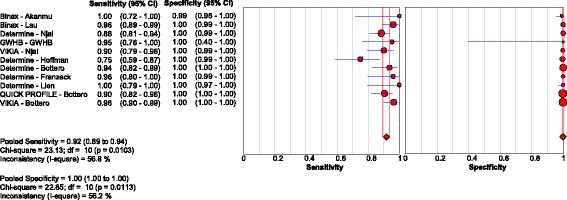
Forest plot with accuracy of RDT compared to EIA, using whole blood only

Most studies used serum or plasma samples. Eight studies had data evaluating five RDTs using capillary or venous whole blood [18, 21, 23, 32, 33, 39, 44, 45], including two that were in exclusively HIV-positive individuals [44, 45]. Pooled sensitivity and specificity in capillary or venous whole blood were comparable to plasma or serum at 91.7% (95% CI: 89.1, 93.9) and 99.9% (95% CI: 99.8, 99.9). Visual and statistical heterogeneity (τ2 = 1.69) were somewhat less among these studies as compared with those described above using a mixture of clinical samples [Fig. 5 **;** Tables 2, 4 and 5].

**Fig. 6:**
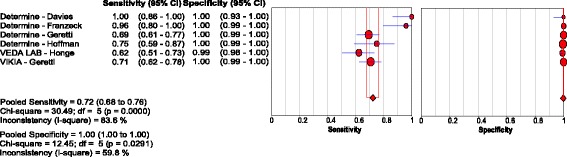
Forest plot with accuracy of RDT compared to EIA, in HIV-positive patients

Five studies [26, 44–47] evaluated three RDTs in 2596 HIV-positive patients, with a pooled sensitivity and specificity of 72.3% (95% CI: 67.9, 76.4) and 99.8% (95% CI: 99.5, 99.9), respectively. Visual and statistical heterogeneity was reduced (τ2 = 1.12). Only one sub-study [18] had extractable data for 224 HIV-negative chronic HBV patients who were HBV treatment naïve [Fig. 6 **;** Tables 2, 4 and 5].

**Fig. 7:**
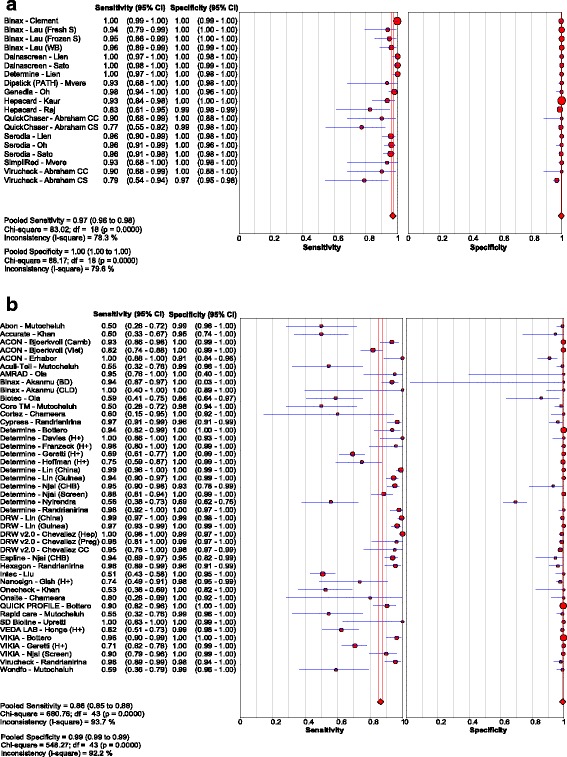
Forest plot with accuracy of RDT compared to EIA, for studies (**a**) before and (**b**) after 2005

Studies published since 2005 reported lower sensitivity compared to the nine articles published before 2005 [20, 27, 30, 32, 33, 36, 38, 40, 42]. Pooled sensitivity was 96.9% (95% CI: 96.0, 97.7) and 86.4% (95% CI: 85.2, 87.5) for studies before and after 2005 respectively [Fig. 7a and b; Table 4]. Five studies [14, 18, 25, 46, 47] published since 2010 evaluating tests against a newer CMIA reference specifically also reported lower pooled sensitivity of 80.4% (95% CI: 77.9, 82.6), with reduced heterogeneity (τ2 = 1.26). Pooled specificity was above 99% irrespective of publication date [Fig. 7b **;** Table 5].

Stratifying by test brand did not substantially reduce heterogeneity. Data for all 50 brands of RDTs and EIAs evaluated [Table 5; Additional file 2] demonstrates broad ranges in sensitivity results within individual brands, with generally high (>90%) specificity, as previously noted. Only four test brands were evaluated in three or more studies. *Determine HBsAg* was evaluated in ten studies, only one published before 2008 [18, 23, 26, 33, 34, 37, 41, 44, 45, 47]; pooled sensitivity and specificity in 7730 samples were 90.8% (95% CI: 88.9, 92.4) and 99.1% (95% CI: 98.9, 99.4), respectively. Excluding one outlier field study that reported a sensitivity of 56% and specificity of 69% [37], the sensitivities ranged from 69% to 100% and specificities from 93% to 100%. *VIKIA HBsAg* was evaluated in three studies in 5242 patient samples [18, 23, 47], all published after 2010, with pooled sensitivity and specificity of 82.5% (95% CI: 77.5, 86.7) and 99.9% (95% CI: 99.8, 100), respectively. *BinaxNOW* HBsAg was evaluated in three studies in 3542 patient samples [21, 27, 32], all published before 2007, with pooled sensitivity and specificity of 97.6% (95% CI: 96.2, 98.6) and 100% (95% CI: 99.7, 100), respectively. *Serodia HBsAg* was evaluated in three studies on 1040 patient samples [33, 38, 42], all published before 2000, with pooled sensitivity and specificity of 95.8% (95% CI: 93.4, 97.5) and 99.8% (95% CI: 99.1, 100), respectively.

### Diagnostic accuracy of laboratory immunoassays for HBsAg detection

**Fig. 8:**
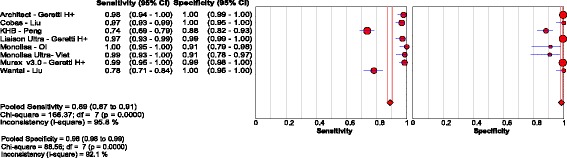
Forest plot with accuracy of EIA compared to EIA

Five studies [14, 47–50], performed in China, Ghana, Cambodia and Vietnam evaluated 8 EIAs against a CMIA reference standard, in 1825 serum or plasma samples, reported a pooled sensitivity and specificity of 88.9% (95% CI: 87.0, 90.6) and 98.4% (95% CI: 97.8, 98.8), respectively. The respective positive and negative LRs were 46.8 (95% CI: 12.9, 170.0) and 0.04 (95% CI: 0.01, 0.13), with visible and statistical heterogeneity between studies (τ2 = 12.00). Outliers were from two Chinese studies [14, 49] that evaluated two older ELISA assays (KHB; Wantai) with a sensitivity lower than 90% [Fig. 8 **;** Tables 1 and 4].

One study [47] evaluated 3 different EIAs in 838 HIV-positive patients. Results were homogenous between tests, with pooled sensitivity and specificity of 97.9% (95% CI: 96.0, 99.0) and 99.4% (95% CI: 99.0, 99.7), respectively, for a positive and negative LR of 167.3 (95% CI: 95.1, 294.1) and 0.02 (95%CI: 0.01, 0.04) respectively [Table 4].

### Diagnostic accuracy compared to a nucleic acid reference standard

#### Rapid diagnostic tests

**Fig. 9:**
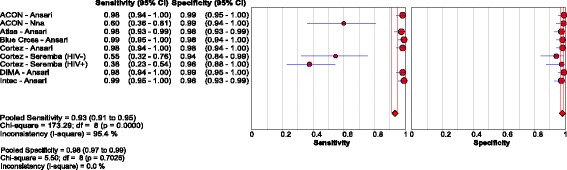
Forest plot with accuracy of RDT compared with NAT

Three studies [51–53] evaluated 7 RDTs in samples from 510 patients against a NAT reference standard, although some samples were used for multiple testing episodes with different tests. One study [52] used plasma from Nigerian repeat blood donors. Sensitivities ranged from 38% to 99% and specificities ranged from 94 to 99%. Overall pooled sensitivity and specificity were 93.3% (95% CI: 91.3, 94.9) and 98.1% (95% CI: 97.0, 98.9), respectively, with significant heterogeneity in terms of sensitivity [Fig. 9 **;** Table 3; Additional file 3]. One case-control study [51] evaluating five different tests in 240 Iranian patients, had significantly higher sensitivity and specificity compared to the other studies, contributing to the overall statistical heterogeneity (τ2 = 5.82).

One study [52] assessed RDT performance in 113 HIV-negative Nigerian repeat blood donors, with clinical sensitivity 60% (95% CI: 36, 81); of note the 8 false negative samples were anti-HBc-positive and regarded as occult hepatitis B, with median HBV viral load 51 IU/ml (range 30–80 IU/mL). The final study [53] had data for consecutive HIV-positive and negative individuals in Uganda; sensitivity was lower in the 83 HIV-positive patients compared to the 74 HIV-negative individuals at 38% (95% CI: 23, 54) and 55% (95% CI: 32, 76) respectively [Table 3; Additional file 3].

#### Enzyme immunoassays

**Fig. 10:**
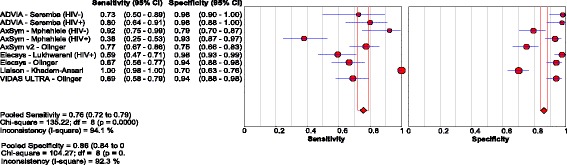
Forest plot with accuracy of EIA compared with NAT

Five studies [53–57] evaluated EIAs based on a NAT reference, using samples from 1194 patients. Pooled sensitivity and specificity were 75.7% (95% CI: 72.1, 79.1) and 86.1% (95% CI: 83.8, 88.2), respectively. The respective positive and negative LRs were 7.2 (95% CI: 4.4, 11.8) and 0.30 (95%CI: 0.19, 0.46), with reduced heterogeneity compared to studies evaluating RDTs (τ2 = 0.90) [Fig. 10 **;** Table 3; Additional file 3].

Three studies [53, 56, 57] had data from 442 HIV-positive patients in Uganda and South Africa, with pooled sensitivity and specificity of 57.9% (95% CI: 49.8, 65.6) and 95.8% (95% CI: 92.7, 97.8), respectively. The corresponding pooled sensitivity and specificity for the 202 HIV-negative patients across two of these studies [53, 57] were 83.3% (95%CI: 69.8, 92.5) and 85.7% (95% CI: 79.2, 90.8), respectively [Table 3; Additional file 3].

## Discussion

### Study findings

Our systematic review and meta-analysis shows that both RDTs and EIAs had excellent specificity for the detection of HBsAg when compared to laboratory-based assays. Although the pooled sensitivity of RDTs was only 90% compared to laboratory based EIAs, the 10% lower sensitivity of RDTs may be an acceptable trade-off for opportunities to use RDTs to increase access to testing to all levels of the health care system. Significant heterogeneity with a broad range of sensitivity estimates was observed across studies and different brands as well as across studies for the same brand. Accuracy and quality of RDTs should be important considerations in test selection for national programmes.

Apart from the rapid results and ease of use, RDTs can be used with whole blood from a finger prick compared to the necessity of processing blood samples to obtain serum or plasma for use with EIAs. Our review showed that accuracy using capillary or venous whole blood was not significantly different from studies using plasma or serum, which offers convenient specimen sampling outside of laboratory settings without compromising test accuracy.

None of the RDTs met minimum requirements for analytical sensitivity (i.e. limit of detection [LOD] of 0.130 IU/mL) required by regulatory authorities such as the European Union; WHO prequalification assessment studies indicate a 50–100 fold lower LoD for EIAs (0.1 IU/mL) compared to RDTs (2–10 IU/mL) [15]. Clinical sensitivity is however unlikely to be greatly reduced as the majority of chronic HBV is associated with blood HBsAg concentrations well above 10 IU/mL and false-negative HBsAg RDTs are associated with lower HBsAg and viral load, presence of HBsAg mutants, or specific genotypes [15, 23, 34, 47].

We found lower sensitivity of RDTs in HIV-positive individuals; however, there did not appear to be a similar reduction in the single study assessing three different EIAs in this cohort using an EIA reference with neutralisation [47]. The reasons for the apparent lower performance are unclear. Studies quantifying HBsAg found that in the context of co-infection, most false negatives had lower concentrations of HBsAg and generally lower HBV DNA than true positives [46, 47]. HIV-reverse transcriptase inhibitors active against HBV can modestly reduce HBsAg levels and therefore detection by RDTs [58–60]; patients treated for a median 47 months demonstrated significantly lower median HBsAg levels compared to untreated patients (3.32log10 vs 4.23log10) (*p* = 0.001), with the most marked reduction in HBeAg positive patients and those with a more robust improvement of CD4 from nadir on cART [61]. In our review, the two studies with preserved sensitivity were in exclusively ART-naïve patients with median CD4 175 cell/uL [26] and 250 cells/uL [44]. Studies with sensitivity less than 80% were in cohorts which included patients on lamivudine-containing ART [46, 47] or ART-naïve with a higher median CD4 (350 cells/uL) [45]. As most patients in the field will be ART-naïve as part of dual screening programmes, the clinical impact of reduced sensitivity could be less significant as most will have detectable higher HBsAg levels. Another theoretical explanation in the context of ART is that given overlapping surface and polymerase genes, lamivudine with its low genetic barrier to resistance could promote the emergence of surface genome variants undetectable by standard assays; mutations in the “a” antigenic determinant region of HBsAg can cause conformational changes leading to decreased accuracy of diagnosis [62]. This was, however, only a minor contributor to reduced performance in the single study assessing mutants in HIV-HBV co-infection [47], with reduced analytical sensitivity of assays more important. Further reasons for reduced sensitivity of lateral flow devices in the context of HIV could be due to either an increased presence of blocking antibodies to HBsAg and immune-complex formation in HIV-associated hypergammaglobulinaemia, or the prozone effect at high antigen concentrations. Assay sensitivity also varies depending on genotypes, and it could be that regions with high HIV co-infection also have a higher proportion of poorly detected genotypes. Finally, as studies were cross-sectional in nature, we can’t assess and compare the true prevalence of chronic HBV in cohorts or the progression of disease – it may be that there is an increased prevalence of acute and/ or chronic HBV in HIV-cohorts, with RDTs missing low level HBsAg in patients who are in the process of seroconverting from their illness. Further studies are required following up patients with HIV and full HBV serology to further ascertain reasons for and the clinical impact of reduced sensitivity of RDTs.

Accuracy of both HBsAg RDTs and EIAs compared to a NAT reference was generally lower, especially amongst HIV-positive cohorts; sensitivity of RDTs was generally <60%, with one laboratory based case-control study evaluating six RDTs contributing to potential over-estimation of pooled sensitivity [51]. Although NAT assays are not optimal reference standards for HBsAg, given the complex relationship between viral kinetics of HBV DNA and levels of HBsAg, NAT assays are nevertheless useful markers of viremia and disease activity to guide treatment, as well as the detection of occult hepatitis B. Occult hepatitis B (OHB) is defined as the presence of HBV DNA in serum or liver tissue with undetectable HBsAg [57]. Studies in ART-naïve East [63] and West-African [64] patients found an OHB prevalence of 10–15%, with significantly lower HBV viral loads in these individuals compared to those with detectable HBsAg [47]. Knowledge of HBeAg status and ART regimes is relevant, as dually active ART could successfully suppress HBV viral load and HBsAg detection [58, 59]. Now that it is possible to use CMIA to quantitate HBsAg, and levels of HBsAg has been correlated with intrahepatic cccDNA clearance during treatment, further research should explore the use of CMIA to quantitate HBsAg levels as potential markers of disease resolution.

The pooled sensitivity for RDTS in this review is lower than that reported in previous systematic reviews (pooled sensitivities were 97.1% [11], 94.8% [10], and 98.1% [9]). This may be due to the use of different inclusion criteria in the prior reviews. Accuracy estimates tend to be higher when the RDTs were evaluated in laboratory settings using archived evaluation panels than when they are evaluated in field settings in patients attending a clinical facility, who may have a variety of underlying conditions or co-infections that affect test performance. In the case of RDTs, the tests may be stored and used in uncontrolled physical environments and performed by users who may not have ever performed a test. Data on the clinical performance of these assays are more relevant for developing guideline recommendations.

### Sources of heterogeneity

Statistical heterogeneity is observed in most diagnostic accuracy reviews. None of the sub-analyses performed eliminated heterogeneity, which could be due to a number of factors. Variability of assays could result in statistical heterogeneity. This persisted despite subgrouping by brand, although it should be noted that the same brand often undergoes minor product changes and modifications over time, particularly with changes in the manufacturer.

Variation in reference standards also contributed to different RDT sensitivity. Pooled sensitivity of RDTs was lower when compared to a CMIA reference standard (80.4%) than a reference including non-CMIA technology (90.0%). ELISA/EIA based assays in particular performed poorly relative to other immunoassays when compared to a CMIA reference [14, 49]; different signal cut-off ratio’s (S/CO) and use of the ‘gray zone’ improved sensitivity at the expense of specificity. Accuracy of tests also varies depending on the phase of chronic HBV infection, with reduced sensitivity more common in the inactive carrier state compared to the active replicative phase. In a Gambian field study [18], the majority (94.7%) of false-negative RDT results were from inactive carriers; they were all HBeAg negative with normal ALT levels, more commonly female (*p* = 0.05) and had lower median quantitative HBsAg levels compared to true positives (1.2 IU/mL vs 875 IU/mL) (*p* = 0.0002). Of note, RDTs also had a lower limit of detection in the field (26.5 IU/mL) compared to the laboratory setting (2.8 IU/mL), although the clinical sensitivity was similar, albeit in a study where field staff were all adequately trained. Although inactive carriers often do not warrant treatment, 17% had elevated liver stiffness and were pre-cirrhotic, so would have benefited from antiviral therapy [65]. Further studies are required to assess the clinical impact of reduced RDT sensitivity, particularly those performed in the field.

Finally, the large variability in study design across the literature is a significant source of heterogeneity. A large number of case-control studies with pre-selection of known cases and controls tend to over-estimate accuracy, in part due to the higher quantitative ranges of HBsAg in those with known active disease. Performance in higher income countries tends to be less heterogeneous [11], while reduced accuracy observed in low-resource settings may be due to insufficient training or lack of quality assurance systems [66]. Pooled sensitivity and specificity tend to be lower when the RDTs are used in the field compared to studies where they were performed in laboratory settings [26, 37].

### Study strengths

Strengths of this review include evaluation of a comprehensive evidence base, use of a pre-specified protocol incorporating numerous major scientific databases, and assessment of additional areas relevant to HBsAg diagnostic testing, notably comparison with NAT and potential impact of occult hepatitis B. We identified 11 additional articles [18, 22, 25, 28, 29, 35, 37, 43, 45–47] not found in the most recent systematic review assessing the diagnostic accuracy of RDTs [11]. The pooled sensitivity for RDTs in this review is lower than reported in previous systematic reviews (pooled sensitivities of 97.1% [11], 94.8% [10], and 98.1% [9]). Potential reasons include the different inclusion criteria; previous reviews included a mixture of studies of analytical performance using serum panels and clinical studies. As previously explained, accuracy estimates tend to be higher when tests are evaluated in laboratory settings using archived evaluation panels, with estimates less relevant for informing the development of testing or operational guidelines.

We included evaluations of both RDTs and EIAs, in addition to evaluation using a NAT reference, and as such are able to evaluate the effects of different types of HBsAg assays and different types of reference assays.

### Limitations

Our study has a number of limitations. Many studies were case-control designs or evaluated cohorts known to over-estimate accuracy. We were unable to assess diagnostic accuracy specifically in field studies as definitions of “in the field” are open to interpretation with methods poorly described in many papers. Only two studies (1, 2) specifically mention the use of RDTs in the field. Since the purpose of our review was to assess clinical performance, we included papers describing evaluations of test performance in patients in clinical settings and not laboratory based evaluations using reference panels. Some analyses were based on a small number of patients and few positive samples. We were unable to explore potential sources of heterogeneity due to genotype, stage and severity of infection or other co-infections; genetic information has long been suspected to impact on diagnostic accuracy [67–72], and mutants are rapidly evolving such that prevalence of specific types cannot be determined on historical data. The use of different reference standards makes pooling across studies difficult; this is further complicated by rapid changes in technology and analytical sensitivity combined with suboptimal reporting of LOD in both index tests and reference standards. For studies using NAT as a reference, assays were not standardized, with poor reporting of testing, albeit all were according to the manufacturer’s instructions; some used pooled NAT of HBsAg negative sample [55, 57], while others described inadequate detail for qPCR methodology [51, 54]. Finally, the natural history of diagnostic markers in chronic hepatitis B is more complex than most viral infections, with transient low level asynchronous quantitative fluctuations of HBsAg and DNA recognised in uncomplicated chronic HBV [73]. Such cases are clinically less severe and of lower priority than persons with higher levels of viremia, but are likely to impact estimates of sensitivity and specificity.

### Implications

The global burden and relative rank of hepatitis B in terms of health loss has increased in the last two decades, unlike most communicable diseases. Implementation of timely and accurate testing strategies in many endemic settings is poor, hindering the linkage to care. Rapid tests are suited to improve the uptake of testing in resource limited settings, particularly amongst remote and vulnerable populations, but evidence is lacking for the impact of testing at the point of care on service delivery and linkage to and uptake of subsequent care. Research is needed on the clinical impact of reduced RDT sensitivity given the association of low quantitative HBsAg missed by testing with inactive carriers and minimal disease progression [74]. Validation of assays in the context of immune escape variants and using less invasive collection methods would support the development of demographic specific testing strategies. Finally, concerns about the low sensitivity of RDTs in HIV positive cohorts warrant particular evaluation, given the growing global challenge posed by co-infection, drug resistance and inadequate approaches to management of HBV and prevention of mother to child transmission in pregnant women [75]. Studies assessing the impact of viral load, CD4 and ART regimen exposure on HBsAg diagnostic accuracy are urgently needed, particularly the potential prudence of repeat HBsAg testing after a certain time in high risk individuals who may have seroconverted or progressed.

## Conclusion

In summary, this meta-analysis demonstrates that RDTs to detect HBsAg, performed on either serum, plasma or whole blood, have a pooled sensitivity of >90% and specificity of >98% compared to laboratory methods of HBsAg detection, using EIAs as the reference standard. Sensitivity varies widely overall and within brands of HBsAg tests. Sensitivity of RDTs may be lower in HIV-positive individuals, although possibly less so in ART-naïve individuals who would benefit most from screening using dual HIV-HBV RDTs in settings with limited access to laboratories. Further research is needed to assess the impact of using RDTs in a variety of settings and populations. WHO guidelines currently recommend a role for RDTs in scaling up HBsAg testing in settings with poor access to or lack of existing laboratory infrastructure, such as remote settings or with hard-to-reach populations. Their use may also be appropriate in high-income countries to increase the uptake of hepatitis testing in populations that may be reluctant to test or have poor access to health-care services and in outreach programmes [12].

## Additional files

Additional file 1: Search strategy. (DOC 80 kb)

Additional file 2: Table S1. Summary pooled diagnostic accuracy of HBsAg assays by brand. (DOC 126 kb)

Additional file 3: Table S2. Summary pooled diagnostic accuracy of HBsAg assays using NAT reference standards. (DOC 75 kb)

## Abbreviations

ART: Anti-retroviral therapy
CC: Case-control studies
CI: Confidence interval
CMIAs: Chemiluminescent microparticle immunoassays
CS: Cross-sectional studies
DBS: Dried blood spot
DOR: Diagnostic odds ratio
ECLIA: Electrochemiluminescent immunoassay
EIA: Enzyme immunoassay
ELISA: Enzyme-linked immunosorbent assay
HBcAb: Hepatitis B core antibody
HBeAg: Hepatitis B ‘e’ antigen
HBsAg: Hepatitis B surface antigen
HBV: Hepatitis B Virus
HCV: Hepatitis C Virus
HIV: Human Immunodefiency virus
LOD: Limit of detection
LR: Likelihood ratio
MEIA: Microparticle enzume immunoassay
NAT: Nucleic acid testing
NLR: Negative likelihood ratio
PLR: Positive likelihood ratio
POC: Point-of-care
PRISMA: Preferred reporting items for systematic reviews and meta-analyses
QUADAS: Quality assessment of diagnostic accuracy studies
RDTs: Rapid diagnostic tests
REM: Random effects model
RIA: Radioimmunoassay
S/CO: Signal cut-off ratio’s
WHO: World Health Organization
