# Supplementary material for: Diagnostic accuracy of tests to detect hepatitis B surface antigen: a systematic review of the literature and meta-analysis
Source: BMC Infect Dis. 2017 Nov 1;17(Suppl 1):698. doi: 10.1186/s12879-017-2772-3 (PMC5688498; doi:10.1186/s12879-017-2772-3)
Supplement: Supplementary file 1 — Search strategy. (DOC 80 kb) [file 12879_2017_2772_MOESM1_ESM.doc]

## Search Strategy

Ovid Medline search strategy

Searched on 20 April 2015 from 1946 – April week 2 2015.

1. Hepatitis, Viral, Human/ (10382)
2. Hepatitis Viruses/ (1363)
3. Hepatitis Antibodies/ (5082)
4. exp Hepadnaviridae Infections/ (47484)
5. Hepatitis B Antibodies/ (8638)
6. Hepatitis B virus/ (20604)
7. Hepadnaviridae/ (192)
8. Hepatitis B Surface Antigens/ (17007)
9. (heptatitis-b or hep-b or (hepatitis adj5 b) or (hep adj5 b) or hbv).ti,ab. (64488)
10. hbsag.ti,ab. (15146)
11. or/1-10 [HEPATITIS B] (87943)
12. exp Reagent Kits, Diagnostic/ (17747)
13. ((rapid or point of care or near patient or poc or poct or bedside) adj5 (test or tests or testing or detect* or diagnos* or screen* or kit or kits or assay* or device*)).ti,ab. (63080)
14. (radt or radts or rdt or rdts).ti,ab. (909)
15. rapid test*.ti,ab. (3400)
16. exp Enzyme-Linked Immunosorbent Assay/ (127391)
17. Immunoassay/ (23237)
18. Immunoenzyme Techniques/ (64864)
19. (enzyme-linked immunosorbent assay or ELISA).ti,ab. (139374)
20. (enzyme adj2 (immunoassay* or immuno-assay* or immunosorbent)).ti,ab. (83849)
21. ((antigen* or antibod*) adj3 detect*).ti,ab. (59427)
22. or/12-21 [RAPID DIAGNOSTIC TESTS] (394724)
23. exp "Sensitivity and Specificity"/ (435087)
24. (diagnos* accura* or sensitiv* or specific* or valid*).ti,ab. (3016884)
25. roc curve.ti,ab. (10226)
26. positive predictive value.ti,ab. (25496)
27. negative predictive value.ti,ab. (20415)
28. or/23-27 [DIAGNOSTIC ACCURACY] (3235789)
29. 11 and 22 and 28 (3103)
30. Humans/ (13846846)
31. Animals/ (5442465)
32. 30 and 31 (1513142)
33. 31 not 32 [ALL ANIMAL STUDIES WHICH DO NOT INCLUDE COMPARISON WITH HUMANS] (3929323)
34. 29 not 33 (2856)
35. limit 34 to english language (2345)

Ovid Embase search strategy

Searched on 20 April 2015 from 1947 – 2015 April 17.

1. hepatitis virus/ (4410)
2. hepatitis antibody/ (2216)
3. exp hepadnaviridae/ (42214)
4. hepatitis B surface antigen/ (27312)
5. (heptatitis-b or hep-b or (hepatitis adj5 b) or (hep adj5 b) or hbv).ti,ab. (94627)
6. hbsag.ti,ab. (22290)
7. or/1-6 [HEPATITIS B] (111321)
8. exp diagnostic kit/ (13384)
9. "point of care testing"/ (5530)
10. ((rapid or point of care or near patient or poc or poct or bedside) adj5 (test or tests or testing or detect* or diagnos* or screen* or kit or kits or assay* or device*)).ti,ab. (88003)
11. (radt or radts or rdt or rdts).ti,ab. (1652)
12. rapid test*.ti,ab. (5049)
13. enzyme linked immunosorbent assay/ (229634)
14. immunoassay/ (48491)
15. enzyme immunoassay/ (36845)
16. enzyme linked immunospot assay/ (6789)
17. enzyme multiplied immunoassay technique/ (768)
18. (enzyme-linked immunosorbent assay or ELISA).ti,ab. (204052)
19. (enzyme adj2 (immunoassay* or immuno-assay* or immunosorbent)).ti,ab. (98704)
20. antigen detection/ (18155)
21. antibody detection/ (34389)
22. ((antigen* or antibod*) adj3 detect*).ti,ab. (76053)
23. or/8-22 [RAPID DIAGNOSTIC TESTS] (525203)
24. "sensitivity and specificity"/ (221828)
25. diagnostic accuracy/ (189329)
26. (diagnos* accura* or sensitiv* or specific* or valid*).ti,ab. (4113104)
27. roc curve.ti,ab. (20232)
28. positive predictive value.ti,ab. (37541)
29. negative predictive value.ti,ab. (31612)
30. or/24-29 [DIAGNOSTIC ACCURACY] (4280338)
31. 7 and 23 and 30 (4018)
32. human/ (15785497)
33. animal/ (1646303)
34. 32 and 33 (404532)
35. 33 not 34 [ALL ANIMAL STUDIES WHICH DO NOT INCLUDE COMPARISON WITH HUMANS] (1241771)
36. 31 not 35 (3963)
37. limit 36 to english language (3344)

Web of Science

Search was conducted on the Science Citation Index Expanded (1970 - 20 April 2015) and the Conference Proceedings Citation Index-Science (1990 - 20 April 2015)

1. TOPIC: ("hepatitis-b" OR "hep-b" OR (hepatitis near/5 b) OR (hep near/5 b) OR hbv) (79,505)
2. TOPIC: (hbsag) (12,160)
3. #2 OR #1 (81,526)
4. TOPIC: ((rapid near/5 test) or (rapid near/5 tests) or (rapid near/5 testing) or (rapid near/5 detect*) or (rapid near/5 diagnos*) or (rapid near/5 screen*) or (rapid near/5 kit) or (rapid near/5 kits) or (rapid near/5 assay*) or (rapid near/5 device*)) (77,863)
5. TOPIC: (("point of care" near/5 test) or ("point of care" near/5 tests) or ("point of care" near/5 testing) or ("point of care" near/5 detect*) or ("point of care" near/5 diagnos*) or ("point of care" near/5 screen*) or ("point of care" near/5 kit) or ("point of care" near/5 kits) or ("point of care" near/5 assay*) or ("point of care" near/5 device*)) (5,974)
6. TOPIC: (("near patient" near/5 test) or ("near patient" near/5 tests) or ("near patient" near/5 testing) or ("near patient" near/5 detect*) or ("near patient" near/5 diagnos*) or ("near patient" near/5 screen*) or ("near patient" near/5 kit) or ("near patient" near/5 kits) or ("near patient" near/5 assay*) or ("near patient" near/5 device*)) (423)
7. TOPIC: ((poc near/5 test) or (poc near/5 tests) or (poc near/5 testing) or (poc near/5 detect*) or (poc near/5 diagnos*) or (poc near/5 screen*) or (poc near/5 kit) or (poc near/5 kits) or (poc near/5 assay*) or (poc near/5 device*)) (866)
8. TOPIC: ((poct near/5 test) or (poct near/5 tests) or (poct near/5 testing) or (poct near/5 detect*) or (poct near/5 diagnos*) or (poct near/5 screen*) or (poct near/5 kit) or (poct near/5 kits) or (poct near/5 assay*) or (poct near/5 device*)) (522)
9. TOPIC: ((bedside near/5 test) or (bedside near/5 tests) or (bedside near/5 testing) or (bedside near/5 detect*) or (bedside near/5 diagnos*) or (bedside near/5 screen*) or (bedside near/5 kit) or (bedside near/5 kits) or (bedside near/5 assay*) or (bedside near/5 device*)) (2,705)
10. TOPIC: (radt or radts or rdt or rdts) (1,406)
11. TOPIC: ("rapid test*") (3,783)
12. TOPIC: ("enzyme-linked immunosorbent assay" or ELISA) (141,435)
13. TOPIC: ((enzyme near/2 immunoassay*) or (enzyme near/2 immuno-assay*) or (enzyme near/2 immunosorbent)) (85,660)
14. TOPIC: ((antigen* near/3 detect*) or (antibod* near/3 detect*)) (56,976)
15. #14 OR #13 OR #12 OR #11 OR #10 OR #9 OR #8 OR #7 OR #6 OR #5 OR #4 (286,936)
16. TOPIC: ("diagnos* accura*" or sensitiv* or specific* or valid*) (4,557,124)
17. TOPIC: ("roc curve") (12,767)
18. TOPIC: ("positive predictive value") (23,706)
19. TOPIC: ("negative predictive value") (18,947)
20. #19 OR #18 OR #17 OR #16 (4,566,667)
21. #20 AND #15 AND #3 (1,789)
22. #20 AND #15 AND #3 Refined by: LANGUAGES: ( ENGLISH ) (1,720)

Scopus

Search was conducted on 20 April 2015

TITLE-ABS-KEY (("heptatitis-b" OR "hep-b" OR (hepatitis W/5 b) OR (hep W/5 b) OR hbv OR hbsag) AND (((rapid OR "point of care" OR "near patient" OR poc OR poct OR bedside) W/5 (tests OR test OR testing OR detect* OR diagnos* OR screen* OR kit OR kits OR assay* OR device*)) OR radt OR radts OR rdt OR rdts OR "rapid test*" OR "enzyme-linked immunosorbent assay" OR elisa OR (enzyme W/2 (immunoassay* OR immuno-assay* OR immunosorbent)) OR ((antibod* OR anigen*) W/3 detect*)) AND ("diagnos* accura*" OR sensitiv* OR specific* OR valid* OR "roc curve" OR "positive predictive value" OR "negative predictive value")) AND (LIMIT-TO (LANGUAGE, "English")) (3,605)

Cochrane Central Register of Controlled Trials, Wiley

The search was run on 20 April 2015

1. MeSH descriptor: [Hepatitis, Viral, Human] this term only
2. MeSH descriptor: [Hepatitis Viruses] this term only
3. MeSH descriptor: [Hepatitis Antibodies] this term only
4. MeSH descriptor: [Hepadnaviridae Infections] explode all trees
5. MeSH descriptor: [Hepatitis B Antibodies] this term only
6. MeSH descriptor: [Hepatitis B virus] this term only
7. MeSH descriptor: [Hepadnaviridae] this term only
8. MeSH descriptor: [Hepatitis B Surface Antigens] explode all trees
9. "hepatitis-b":ti,ab,kw (Word variations have been searched)
10. "hep-b":ti,ab,kw (Word variations have been searched)
11. hepatitis near/5 b:ti,ab,kw (Word variations have been searched)
12. hep near/5 b:ti,ab,kw (Word variations have been searched)
13. hbv:ti,ab,kw (Word variations have been searched)
14. hbsag:ti,ab,kw (Word variations have been searched)
15. #1 or #2 or #3 or #4 or #5 or #6 or #7 or #8 or #9 or #10 or #11 or #12 or #13 or #14
16. MeSH descriptor: [Reagent Kits, Diagnostic] explode all trees
17. (rapid or "point of care" or "near patient" or poc or poct or bedside) near/5 (test or tests or testing or detect* or diagnos* or screen* or kit or kits or assay* or device*):ti,ab,kw (Word variations have been searched)
18. radt or radts or rdt or rdts:ti,ab,kw (Word variations have been searched)
19. "rapid test*":ti,ab,kw (Word variations have been searched)
20. MeSH descriptor: [Enzyme-Linked Immunosorbent Assay] explode all trees
21. enzyme near/2 (immunoassay* or immuno-assay* or immunosorbent):ti,ab,kw (Word variations have been searched)
22. (antigen* or antibod*) near/3 detect*:ti,ab,kw (Word variations have been searched)
23. MeSH descriptor: [Immunoassay] this term only
24. MeSH descriptor: [Immunoenzyme Techniques] this term only
25. "enzyme-linked immunosorbent assay" or ELISA:ti,ab,kw (Word variations have been searched)
26. #16 or #17 or #18 or #19 or #20 or #21 or #22 or #23 or #24 or #25
27. MeSH descriptor: [Sensitivity and Specificity] explode all trees
28. diagnos* accura* or sensitiv* or specific* or valid*:ti,ab,kw (Word variations have been searched)
29. "roc curve":ti,ab,kw (Word variations have been searched)
30. "positive predictive value":ti,ab,kw (Word variations have been searched)
31. "negative predictive value":ti,ab,kw (Word variations have been searched)
32. #27 or #28 or #29 or #30 or #31
33. #15 and #26 and #32

The search found 64 trials.

Literatura Latino-Americana e do Caribe em Ciências da Saúde (LILACS) (BIREME interface)

LILACS was searched on 20 April 2015

("hepatitis b" or "hep b" or "hbv" or "hbsag") and ("rapid test$" or "point of care test$" or "near patient test$" or "poc test$" or poct or "bedside test$" or "rapid detect$" or "point of care detect$" or "near patient detect$" or "poc detect$" or "bedside detect$" or "rapid diagnos$" or "point of care diagnos$" or "near patient diagnos$" or "poc diagnos$" or "bedside diagnos$" or "rapid screen$" or "point of care screen$" or "near patient screen$" or "poc screen$" or "bedside screen$" or "rapid kit$" or "point of care kit$" or "near patient kit$" or "poc kit$" or "bedside kit$" or "rapid assay$" or "point of care assay$" or "near patient assay$" or "poc assay$" or "bedside assay$" or "rapid device$" or "point of care device$" or "near patient device$" or "poc device$" or "bedside device$" or radt or radts or rdt or rdts or "enzyme-linked immunosorbent assay" or "antigen$ detect$" or "antibod$ detect$" or elisa or immunoassay or immunoenzyme or "immuno-assay") and ("diagnos$ accura$" or sensitiv$ or specific$ or valid$ or "roc curve" or "positive predictive value" or "negative predictive value") (33)

WHO Global Index Medicus

The database was searched on 22 April 2015

SUBJECT: (("Hepatitis, Viral, Human" OR "Hepatitis Viruses" OR "Hepatitis B virus" OR "Hepatitis Antibodies" OR "Hepadnaviridae Infections" OR "Hepatitis B Antibodies" OR "Hepatitis B Virus" OR "Hepadnaviridae" OR "Hepatitis B Surface Antigens") AND ("Reagent Kits, Diagnostic" OR "Enzyme-Linked Immunosorbent Assay" OR "Immunoassay" OR "Immunoenzyme Techniques") AND ("Sensitivity and Specificity")) (478)
