# Supplementary material for: Diagnostic accuracy of tests to detect hepatitis B surface antigen: a systematic review of the literature and meta-analysis
Source: BMC Infect Dis. 2017 Nov 1;17(Suppl 1):698. doi: 10.1186/s12879-017-2772-3 (PMC5688498; doi:10.1186/s12879-017-2772-3)
Supplement: Supplementary file 2 — Summary pooled diagnostic accuracy of HBsAg assays by brand. (DOC 126 kb) [file 12879_2017_2772_MOESM2_ESM.doc]

## Table S1. Summary pooled diagnostic accuracy of HBsAg assays by brand.

| ***Test***  ***Type*** | **Brand Name** | **Studies, n**  **(Data points, n)** | **Reference (EIA)** | | **Studies, n**  **(Data points, n)** | **Reference (NAT)** | |
| --- | --- | --- | --- | --- | --- | --- | --- |
|  |  |  | ***Sen (95% CI)*** | ***Spec (95% CI)*** |  | ***Sen (95% CI)*** | ***Spec (95% CI)*** |
| **RDT** | Abon | 1 (1) | 50.0 (28.2-71.8) | 99.2 (95.7-100) | ND | ND | ND |
| **RDT** | Accurate | 1 (1) | 50.0 (33.4-66.6) | 94.7 (74.0-99.9) | ND | ND | ND |
| **RDT** | ACON | 2 (3) | 88.0 (83.4-91.7) | 99.4 (99.0-99.7) | 2 (2) | 92.9 (87.3-96.5) | 99.1 (96.6-99.9) |
| **RDT** | Acull-Tell | 1 (1) | 54.5 (33.2-75.6) | 99.2 (95.7-100) | ND | ND | ND |
| **RDT** | AMRAD | 1 (1) | 95.2 (76.2-99.9) | 100 (39.8-100) | ND | ND | ND |
| **RDT** | Atlas | ND | ND | ND | 1 (1) | 97.5 (92.9-99.5) | 97.5 (92.9-99.5) |
| **RDT** | BinaxNOW | 3 (6) | 97.6 (96.2-98.6) | 99.9 (99.7-100) | ND | ND | ND |
| **RDT** | Blue Cross | ND | ND | ND | 1 (1) | 99.2 (95.4-100) | 98.3 (94.1-99.8) |
| **RDT** | Biotec | 1 (1) | 58.8 (40.7-75.4) | 85.7 (63.7-97.0) | ND | ND | ND |
| **RDT** | Core TM | 1 (1) | 50.0 (28.2-71.8) | 98.4 (94.5-99.8) | ND | ND | ND |
| **RDT** | Cortez | 1 (1) | 60.0 (14.7-94.7) | 100 (92.1-100) | 2 (3) | 79.7 (73.1-85.3) | 97.2 (94.0-99.0) |
| **RDT** | Cypress | 1 (1) | 96.7 (90.7-99.3) | 96.3 (90.9-99.0) | ND | ND | ND |
| **RDT** | Dainascreen | 2 (2) | 100 (98.7-100) | 100 (99.3-100) | ND | ND | ND |
| **RDT** | Determine | 10 (12) | 90.8 (88.9-92.4) | 99.1 (98.9-99.4) | ND | ND | ND |
| **RDT** | DIMA |  |  |  | 1 (1) | 98.3 (94.1-99.8) | 99.2 (95.4-100) |
| **RDT** | Dipstick (PATH) | 1 (1) | 93.3 (68.1-99.8) | 100 (98.1-100) | ND | ND | ND |
| **RDT** | DRW | 1 (2) | 98.1 (96.1-99.2) | 99.5 (98.8-99.9) | ND | ND | ND |
| **RDT** | DRW v2 | 1 (3) | 99.3 (97.4-99.9) | 98.3 (97.5-98.9) | ND | ND | ND |
| **RDT** | Espline | 1 (1) | 93.9 (89.1-97.1) | 94.7 (82.3-99.4) | ND | ND | ND |
| **RDT** | Genedia | 1 (1) | 98.0 (94.3-99.6) | 100 (96.4-100) | ND | ND | ND |
| **RDT** | Hepacard | 2 (2) | 90.5 (82.1-95.8) | 99.7 (99.5-99.9) | ND | ND | ND |
| **RDT** | Hexagon | 1 (1) | 95.6 (89.1-98.8) | 96.4 (90.9-99.0) | ND | ND | ND |
| **RDT** | Intec | 1 (1) | 50.8 (43.3-58.4) | 100 (94.9-100) | 1 (1) | 99.2 (95.4-100) | 97.5 (92.9-99.5) |
| **RDT** | Nanosign | 1 (1) | 73.7 (48.8-90.9) | 97.8 (95.4-99.2) | ND | ND | ND |
| **RDT** | Onecheck | 1 (1) | 52.6 (35.8-69.0) | 100 (82.4-100) | ND | ND | ND |
| **RDT** | Onsite | 1 (1) | 80.0 (28.4-99.5) | 100 (92.1-100) | ND | ND | ND |
| **RDT** | Quick Profile | 1 (1) | 90.5 (82.1-95.8) | 99.7 (99.5-99.9) | ND | ND | ND |
| **RDT** | QuickChaser | 1 (2) | 83.3 (68.6-93.0) | 99.5 (98.2-99.9) | ND | ND | ND |
| **RDT** | Rapid Care | 1 (1) | 54.5 (32.2-75.6) | 99.2 (95.7-100) | ND | ND | ND |
| **RDT** | SD Bioline | 1 (1) | 100 (63.1-100) | 100 (98.9-100) | ND | ND | ND |
| **RDT** | Serodia | 3 (3) | 95.8 (93.4-97.5) | 99.8 (99.1-100) | ND | ND | ND |
| **RDT** | SimpliRed | 1 (1) | 93.3 (68.1-99.8) | 100 (98.1-100) | ND | ND | ND |
| **RDT** | VEDA Lab | 1 (1) | 62.3 (50.6-73.1) | 99.2 (97.6-99.8) | ND | ND | ND |
| **RDT** | VIKIA | 3 (3) | 82.5 (77.5-86.7) | 99.9 (99.8-100) | ND | ND | ND |
| **RDT** | Virucheck | 2 (3) | 92.3 (86.3-96.2) | 97.3 (95.5-98.5) | ND | ND | ND |
| **RDT** | Wondfo | 1 (1) | 59.1 (36.4-79.3) | 99.2 (95.7-100) | ND | ND | ND |
| **EIA** | ADVIA | ND | ND | ND | 1 (2) | 77.4 (65.0-87.1) | 97.9 (92.6-99.7) |
| **EIA** | Architect | 1 (1) | 97.9 (93.9-99.6) | 99.6 (98.7-99.9) | ND | ND | ND |
| **EIA** | AxSym | ND | ND | ND | 1 (2) | 56.6 (44.7-67.9) | 86.8 (81.5-90.9) |
| **EIA** | AxSym v2 | ND | ND | ND | 1 (1) | 77.4 (67.0-85.8) | 75.0 (66.1-82.6) |
| **EIA** | Cobas | 1 (1) | 96.6 (92.8-98.8) | 100 (94.9-100) | ND | ND | ND |
| **EIA** | Elecsys | ND | ND | ND | 2 (2) | 63.4 (55.2-71.0) | 95.8 (92.4-98.0) |
| **EIA** | KHB | 1 (1) | 73.8 (68.7-78.6) | 88.1 (82.4-92.5) | ND | ND | ND |
| **EIA** | Liaison | ND | ND | ND | 1 (1) | 100 (97.6-100) | 70.0 (63.1-76.3) |
| **EIA** | Liaison Ultra | 1 (1) | 97.1 (92.8-99.2) | 99.4 (98.5-99.8) | ND | ND | ND |
| **EIA** | Monolisa | 1 (1) | 100 (95.2-100) | 91.1 (78.8-97.5) | ND | ND | ND |
| **EIA** | Monolisa Ultra | 1 (1) | 98.7 (92.9-100) | 90.7 (77.9-97.4) | ND | ND | ND |
| **EIA** | Murex v3 | 1 (1) | 98.6 (94.9-99.8) | 99.3 (98.3-99.8) | ND | ND | ND |
| **EIA** | VIDAS Ultra | ND | ND | ND | 1 (1) | 69.0 (58.0-78.7) | 94.0 (88.0-97.5) |
| **EIA** | Wantai | 1 (1) | 78.2 (71.4-84.0) | 100 (94.9-100) | ND | ND | ND |

ND = No Data
