# Supplementary material for: Diagnostic accuracy of tests to detect hepatitis B surface antigen: a systematic review of the literature and meta-analysis
Source: BMC Infect Dis. 2017 Nov 1;17(Suppl 1):698. doi: 10.1186/s12879-017-2772-3 (PMC5688498; doi:10.1186/s12879-017-2772-3)
Supplement: Supplementary file 3 — Summary pooled diagnostic accuracy of HBsAg assays using NAT reference standards. (DOC 75 kb) [file 12879_2017_2772_MOESM3_ESM.doc]

Table S2. Summary pooled diagnostic accuracy of HBsAg assays using NAT reference standards.

| **Index Test** | **HIV Status** | **Studies, n** | **Sample size (range), n** | **Data points, n** | **Pooled clinical accuracy (95% CI)** | | **Likelihood Ratios (95% CI)** | |
| --- | --- | --- | --- | --- | --- | --- | --- | --- |
|  |  |  |  |  | *Sensitivity* | *Specificity* | *Positive* | *Negative* |
| ***RDT*** | NA | 3 | 510  (113-157) | 9 | 93.3  (91.3-94.9) | 98.1  (97.0-98.9) | 39.4  (22.1-70.2) | 0.05  (0.01-0.27) |
|  | HIV-positive | 1 | 83 | 1 | 37.5  (22.7-54.2) | 97.7  (87.7-99.9) | ND | ND |
|  | HIV-negative | 2 | 187  (74-113) | 2 | 57.1  (41.0-72.3) | 97.2  (93.1-99.2) |  |  |
| ***EIA*** | NA | 5 | 1194  (157-350) | 9 | 75.7  (72.1-79.1) | 86.1  (83.8-88.2) | 7.2  (4.4-11.8) | 0.30  (0.19-0.46) |
|  | HIV-positive | 3 | 442  (83-192) | 3 | 57.9  (49.8-65.6) | 95.8  (92.7-97.8) | 14.4  (3.8-55.3) | 0.41  (0.23-0.73) |
|  | HIV-negative | 2 | 202  (74-128) | 2 | 83.3  (69.8-92.5) | 85.7  (79.2-90.8) | 11.3  (0.8-149.8) | 0.19  (0.06-0.56) |
